# Cardiovascular outcomes following Kawasaki disease in Moscow, Russia: A single center experience

**DOI:** 10.21542/gcsp.2017.23

**Published:** 2017-10-31

**Authors:** Galina Lyskina, Olga Bockeria, Olga Shirinsky, Alena Torbyak, Anna Leontieva, Nina Gagarina, Anna Satyukova, Julia Kostina, Olga Vinogradova

**Affiliations:** 1 I.M.Sechenov First Moscow State Medical University, Moscow; 2 Bakoulev Scientific Center for Cardiovascular Surgery RAMS, Moscow

**Keywords:** Kawasaki syndrome, Kawasaki disease, Coronary artery aneurysms, Coronary artery thrombosis, Coronary artery stenosis

## Abstract

**Background:** Awareness of Kawasaki disease (KD) is emerging in Russia but the diagnosis is still often missed.

**Methods:** This is a retrospective study of 303 children with KD who received care at a single center in Moscow over the period from 2004 to 2016.

**Results:** Overall, coronary artery aneurysms were documented in 91 (30,0%) of 303 patients and transient ectasia in 40 (13,2%). Intracoronary thrombi were found in 12 of 15 patients with giant aneurysms and in 3 patients with medium-sized aneurysms.

**Conclusion:** The patients with KD in the Moscow region had typical features of the disease described in the literature but the proportion of patients with coronary artery aneurysms was higher than reported from other countries. We assume that this is due to delayed treatment, which has gradually improved over time. Increased awareness of KD in Russia is critical to ensure timely diagnosis and treatment.

## Introduction

Timely diagnosis and treatment with intravenous immunoglobulin are necessary to reduce the incidence of cardiovascular complications in patients with Kawasaki disease (KD)^1–3^. However, early diagnosis is problematic because there are no pathognomonic clinical signs or specific diagnostic tests. In Russia, diagnosis is complicated by the lack of physicians’ awareness about KD. Thus, it can be assumed that many patients with KD are never diagnosed and the true incidence of disease in Russia is unknown.

It is necessary to improve the system of long-term management of KD^4–6^. For this purpose, it may be useful to accumulate the experience and to analyze aspects of the management of patients with KD in different countries. Here we describe the outcomes of patients diagnosed with KD in the Moscow region.

## Materials and Methods

This is a retrospective study of 303 children with KD who received care at the University Children’s Hospital of I.M.Sechenov First Moscow State Medical University over the period from 2004 to 2016. Some patients were referred to our clinic for examination and treatment from other regions of Russia.

The follow-up period after KD onset was from 0.42 to 13 years. Serial evaluation of patients with coronary artery aneurysms included clinical examination, ECG, transthoracic echocardiography every 3–12 months depending on the severity of the coronary lesion. Stress ECG was performed in patients older than 4–5 years with medium and giant aneurysms every 1–2 years.

Invasive coronary angiography was performed in 14 patients in B.V. Petrovsky Russian Scientific Surgery Center and in A.N. Bakoulev Scientific Center for Cardiovascular Surgery. Multidetector computed tomography (MDCT) of coronary arteries was applied in 32 children. MDCT was performed on a 320-spiral tomograph Toshiba Aquilion ONE, Toshiba, Japan. Examination was performed in a volume mode (without table moving, pitch = 0) with a slice thickness of 0.5 mm and further reconstruction up to 0.25 mm. Voltage parameters depended on the patient’s body weight (100–250 mA), tube current −80–100 kV. Study protocol included examination without contrast media for accurate determination of scan area and determination of the coronary calcium score and further examination with contrast agent injection.

Non-ionic contrast agent (iodixanol or iohexol) was administered through an antecubital catheter via automatic injector at a rate of 2.5–4 mL/sec. The amount of contrast agent was from 20 to 40 ml and depended on the patient’s body weight (not more than 1.5 ml/kg).

Contrast phase of study was started from the level of 10 mm above left main coronary artery origin to cardiac apex and posterior wall of left ventricle. We used a prospective synchronization with ECG with an extended interval (45–75% of R-R interval) to assess coronary arteries during systole and diastole. Post-processing included the assessment of coronary calcification with calculation of plaque volume and calcium score by Agatston, plotting of multiplanar reformations and three-dimensional reconstructions. Total time of the study did not exceed 10 minutes and the scan time was not more than 0,45 sec, so patients did not require sedation in most cases. Premedication was used in young children (up to 3 years). The radiation dose in the study did not exceed 1 mSv.

The main method for coronary artery visualization was echocardiography because it is noninvasive, readily available, has no radiation exposure and may be repeated many times. Echocardiography was performed in all children on the Vivid 5 and Vivid 9 (GE, Germany) using a multi-frequency transducers with a frequency of 5 to 8 MHz. In addition to standard studies specific techniques for visualization of left coronary artery trunk (LCA), left anterior descending (LAD) and circumflex artery, proximal, middle and distal segments of right coronary artery (RCA), posterior descending artery were used^7^.

We measured the inner diameter of coronary arteries. Lumen diameter and external diameter of aneurysm were measured if there were significant coronary artery wall thickening or mural thrombi. Coronary artery aneurysms’ criteria: local vessel extension diameter of ≥2,5 Z-scores or if it exceeded ≥1,5 times the diameter of the adjacent segment. Aneurysms were classified as follows: Small aneurysms <5 mm, medium 5-8 mm, giant >8 mm.^2^ Coronary artery ectasia was considered transient if it disappeared within 8 weeks after KD onset.

### Ethics approval

The study was approved by I.M.Sechenov First Moscow State Medical University’s ethics committee.

### Statistical analysis

Data are presented as medians (IQR). Differences were tested using the Mann–Whitney test, Fisher’s exact test, chi-square, Student’s *t*-test. A two-tailed *P*-value of less than 0.05 was considered significant. IBM SPSS Statistics, version 22 for Windows, was used for all statistical analysis.

**Table 1:** Patient characteristics.

Characteristics	Cases (total *n* = 303)
Gender: male/female: *n* (%)	184 (60,7)/119(39,3)
Age at onset of KD (yrs): median (IQR)	1,6 (2,4)
Complete/incomplete KS: %	86,4/13,6
Treatment in acute stage:	
IVIG + acetylsalicylic acid: *n* (%)	240 (79,2)
no IVIG: *n* (%)	29 (9,5)
no information: *n* (%)	34 (11,2)
Treatment in the first 7 days from onset of KS: *n* (%)	68 (22,4)
Treatment on the 8th–10th day from onset of KS: *n* (%)	67 (22,3)
Treatment after the 10th day from onset of KS: *n* (%)	105 (34,6)

## Results

**Figure 1.**
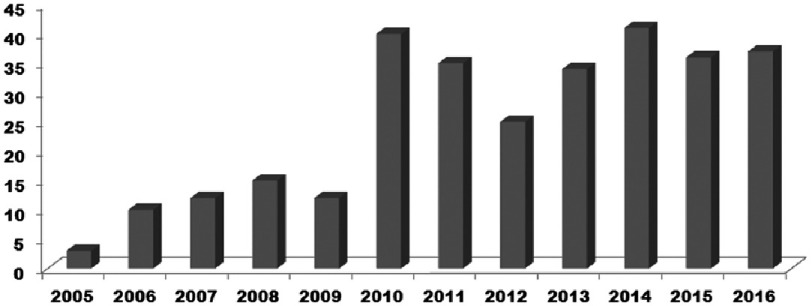
Cases of KS admitted to University Children’s Hospital of I.M.Sechenov First Moscow State Medical University by year.

The demographic and clinical features of our study cohort are summarized in Table 1. For the period from 2004 to 2006 there were sporadic patients with KD in our hospital. From 2010 to 2016 the number of new cases of KS rose to 30 to 40 per year, which likely reflects increased awareness of KD in the Moscow region (Figure 1).

**Figure 2.**
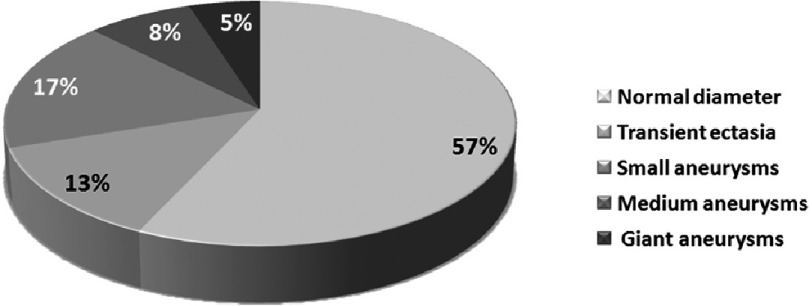
Coronary artery outcomes by echocardiography.

Coronary artery aneurysms were documented in 91 (30,0%) of 303 patients and transient ectasia in 40 (13,2%) (Figure 2). The incidence of coronary artery aneurysms in infants was higher than in children older than one year, 38,8% (38 of 98) and 24,9% (52 of 205), respectively *P* < 0, 05. Most aneurysms (72.4%) were located in the proximal segments of coronary arteries with 52.8% in right coronary artery and 47.2% in the left coronary artery and its branches.

**Figure 3.**
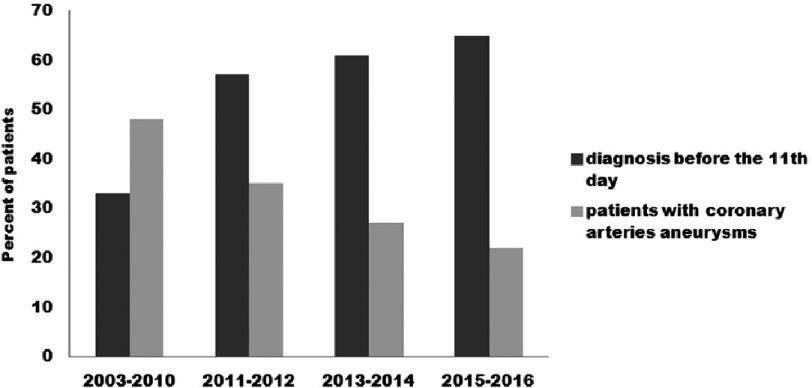
Timing of KD patient diagnosis and treatment and the effect on aneurysm formation.

During the study period, the number of patients diagnosed within the first 10 days of illness increased, while the incidence of coronary aneurysms decreased (Figure 3) and the severity of coronary artery lesions decreased. In 2016 we detected the smallest frequency of coronary artery aneurysms’ formation, there were no giant aneurysms, medium-sized aneurysms were found only in one patient.

### Regression of aneurysms

**Figure 4.**
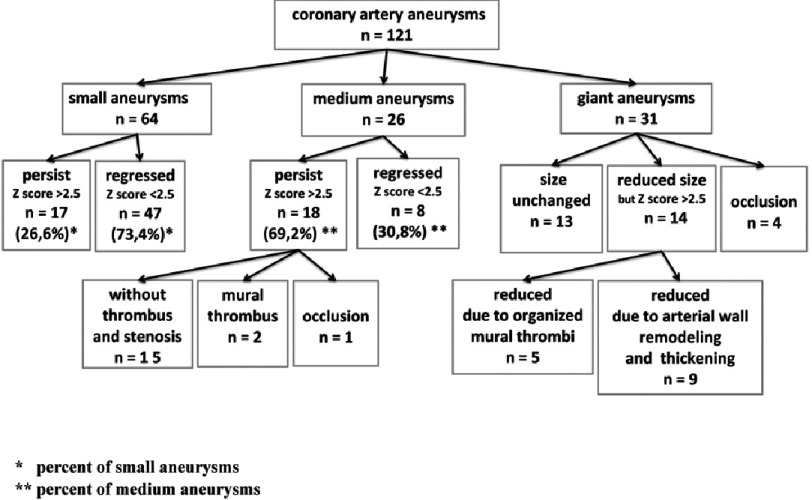
Fate of aneurysms, depending on their size.

The regression of coronary artery aneurysms and the relationship to aneurysm size were studied in 58 patients who developed aneurysms and were under observation at least one year after KD onset. Overall, there were 121 aneurysms. The fate of aneurysms is shown in Figure 4. Lumen diameter of aneurysms decreased due to arterial wall remodeling coupled with organized mural thrombi. In other cases we observed significant reduction of aneurysm size without any noticeable thickening of the coronary artery wall.

### Characteristics of patients with giant aneurysms

**Figure 5.**
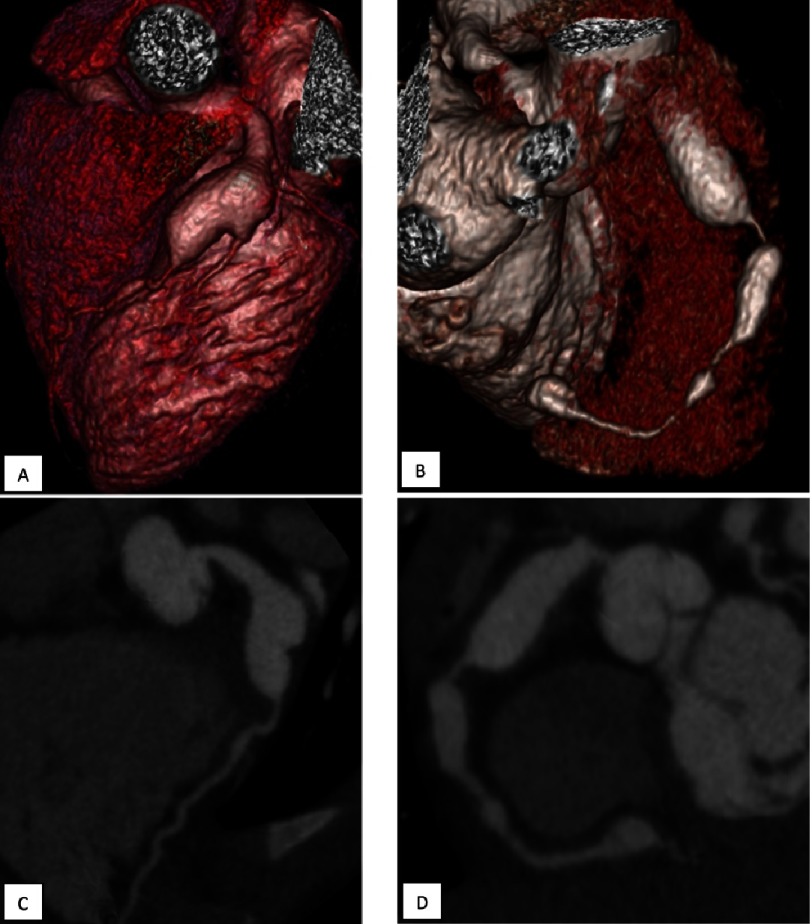
Giant aneurysms of the coronary arteries in a boy who suffered KS at the age of 1,25 years. The diagnosis of KS was made and the IVIG 2 g/kg was administered on the 12-th day of illness. The maximum diameter of the aneurysm of the LCA 12 mm, 31,6 Z-score (A, C), RCA – 9 mm 19,9 Z-score (B, D). MDCT, A and B - three-dimensional reconstructions, C and D - multiplanar reformation.

**Figure 6.**
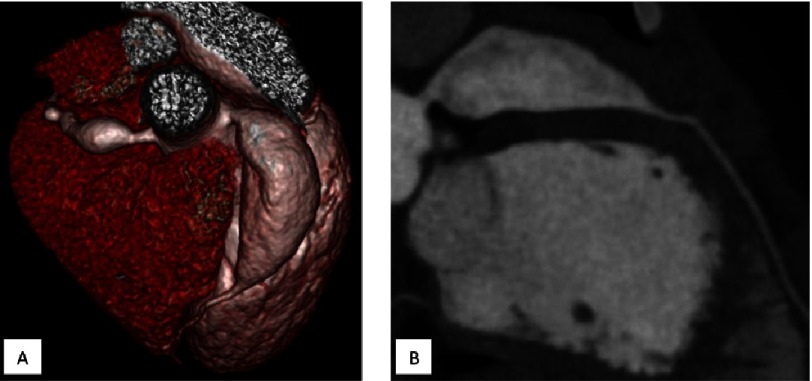
The biggest aneurysm in our cohort in a boy who suffered KS at the age of 4 years. The diagnosis of KS was made and the IVIG 2 g/kg was administered on the 24-th day of illness. The diameter of the LAD aneurysm was 17 mm, 42,25 Z-score, A - three-dimensional reconstructions, B - multiplanar reformation.

**Figure 7.**
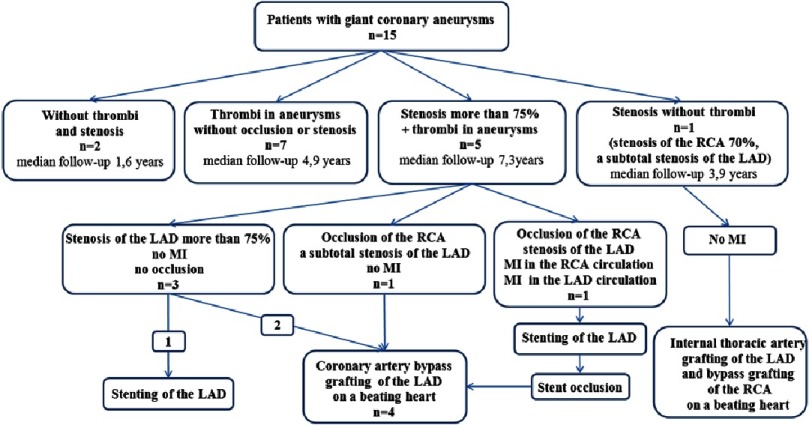
KS outcomes in 15 patients with giant aneurysms of the coronary arteries. RCA, right coronary artery; LAD, left anterior descending artery; MI, myocardial infarction.

Giant aneurysms in our study cohort were observed only in patients who received intravenous immunoglobulin after 10 days of illness and were more common in males (Figures 5 and 6). Characteristics of patients with giant aneurysms are presented in the Table 2*.* The detection frequency of giant aneurysms in RCA and LCA was equal. 24 giant aneurysms were found in proximal segments of coronary arteries, 7 were found in middle ones, 2 were located in distal ones. All patients except one had not only giant aneurysms but also aneurysms of smaller diameter in other segments of coronary arteries. Large intracoronary thrombi, occlusion, and severe stenosis of the coronary arteries were found mostly in patients with giant aneurysms. The outcomes of giant aneurysms are shown in Figure 7.

**Table 2:** Characteristics of 15 patients who developed giant aneurysms.

Gender: male/female: *n*		12/3
Age at onset of KD (yrs): median (IQR)		1,6 (2,4)
Median Interval from the onset to diagnosis (IQR) (days)		24 (10,5)
Median follow-up period from the KS onset (IQR) (yrs)		4,33 (5,6)
Maximal diameter of aneurysm, mm/Z score		17/42,25
Localization of the giant aneurysms LCA/RCA/LCA + RCA		4∕3∕8
Amount of giant aneurysms in one patient, median (min – max)		2 (1 – 5)
Change in giant aneurysms over time (*n* = 31)	no change	13
	Z score reduced but >2.5	14
	Z score < 2.5	0
	Occlusion	4
Thrombotic lesions	Localization of the thrombi LCA/RCA/LCA + RCA	6∕4∕2
	Median interval from the onset to detection of thrombosis (IQR) (yrs)	0,17 (0,29)
Stenotic lesions more than 75% *n* = 5	Localization of the stenosis LCA/RCA/LCA + RCA	4∕0∕1
	Median Interval from the onset to detection of stenosis (IQR) (yrs)	2,5 (0,17)
Surgical treatment *n* = 5	Median Interval from the onset to surgical treatment (IQR) (yrs)	2,8 (0,6)
	Patient’s age at the time of surgical treatment (yrs), median (min – max)	4,58 (3 – 13,75)

**Notes.**

LMCA Left main coronary artery LAD Left anterior descending artery RCA Right coronary artery

**Figure 8.**
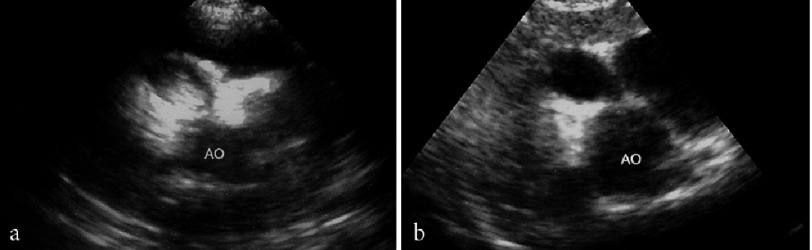
Blood clot regression in giant aneurysm of right coronary artery. a - aneurysm of proximal segment of right coronary artery with a diameter of 10 mm and large thrombus was revealed using echocardiography first performed in 2 months after KS onset, b – in 3 months thrombus in the aneurysm is not visualized.

**Figure 9.**
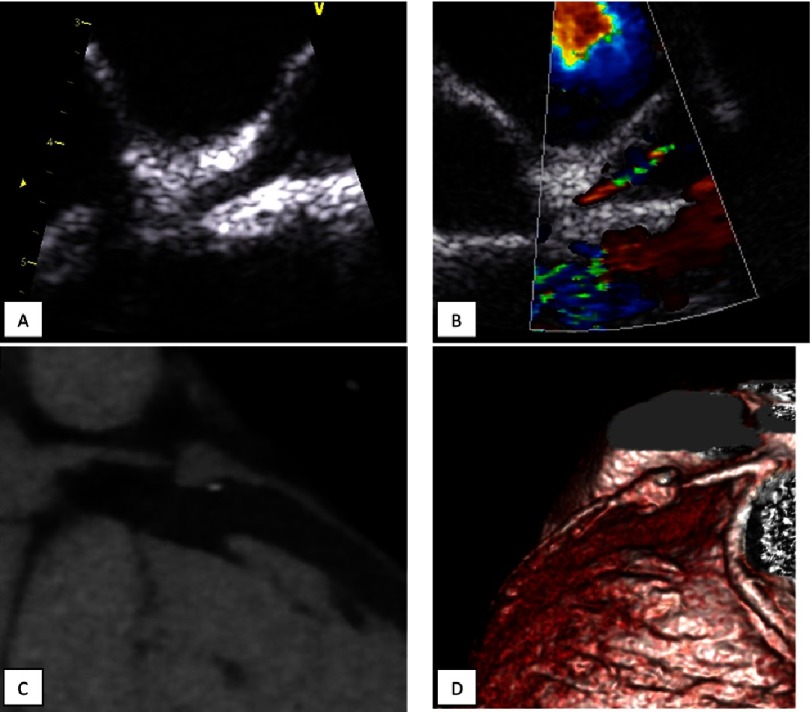
A subtotal stenosis at the entrance of an aneurysm of the LAD 2,67 years after the onset of KS in a boy who suffered KS at the age of 1,58 years. A – echocardiogram. A significant thickening of aneurysmal walls in LAD especially at its entrance. B – echocardiogram. Aliasing phenomenon can be seen at the entrance of an aneurysm in color flow mode that reflects a high-speed flow. The flow rate in LAD before the aneurysm in PW mode was 0,4 m/s while at the entrance of an aneurysm it exceeded 1,5 m/s. C, D - MDCT, C - multiplanar reformation, D - three-dimensional reconstruction. A subtotal stenosis at the entrance of an aneurysm. Calcification can be seen in the walls of an aneurysm.

**Figure 10.**
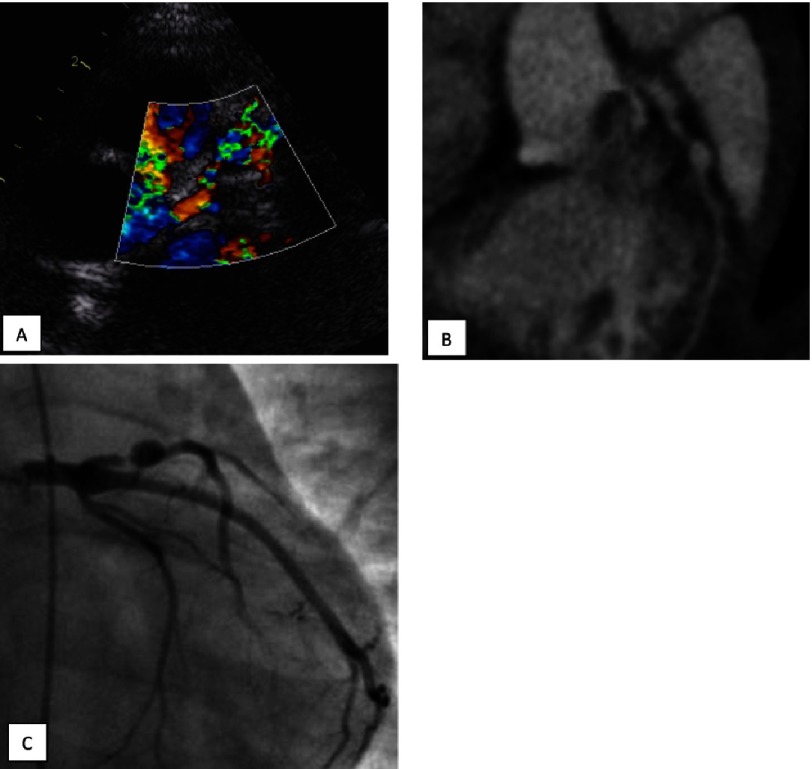
A subtotal stenosis at the entrance of an aneurysm of the LAD 2,5 years after the onset of KS in a boy who suffered KS at the age of 0,42 years. A – echocardiogram, color flow mode. B – MDCT, multiplanar reformation, C - invasive X-ray coronary angiography.

### Coronary thrombosis

Intracoronary thrombi were found in 12 of 15 patients with giant aneurysms and in 3 patients with medium-sized aneurysms. Several thrombi regressed during the follow-up without thrombolysis while using the standard antithrombotic therapy with heparin, low-molecular wieght heparins, warfarin, and aspirin (Figure 8). The majority of mural thrombi organized.

Obstruction of the right coronary artery developed in two patients with giant aneurysms and in one patient with medium-sized aneurysms in spite of prophylaxis for thrombosis (patient 1–aspirin + clopidogrel + heparin/fragmin, patient 2—aspirin + warfarin, patient 3—aspirin). The clinical consequences of right coronary artery occlusion were different in our patients: in two of them there were no clinical signs of ischemia; in the third patient the occlusion of the right coronary artery led to the myocardial infarction of the basal posterior segments of the left ventricle 33 days after KD onset. Three months later, this patient suffered from a repeated myocardial infarction as a result of the massive blood clot and critical stenosis of the middle segment of the left anterior descending coronary artery.

### Coronary stenosis

Coronary artery stenosis more than 75% was found in 5 patients in the entrance or exit of giant aneurysms, 3 of these patients had subtotal stenosis (Figures 9, 10). In 3 patients the stenosis progression and the significant reduction of giant aneurysms’ diameter developed simultaneously due to thickening of the coronary artery wall. Chest pain and ECG changes were present only in 1 patient with myocardial infarction. 4 of the 5 patients had no complaints and no evidence of ischemia according to ECG and Holter monitoring.

Treadmill stress test performed in one case (other patients were younger than 4 years) showed no latent ischemia. Echocardiography demonstrated the presence of hemodynamically significant stenosis in 3 patients in the inlet of the aneurysm at the proximal segment of the LAD artery that was confirmed by CT and angiography (Figures 8 and 9). Stenosis in the outlet of aneurysms of the LAD and stenosis of the RCA were detected using CT and angiography).

Five patients underwent surgical treatment in view of the severe stenosis of the left anterior descending artery. Minimally invasive coronary revascularization - stent placement or mammary artery bypass grafting of LAD on a beating heart were performed. One patient suffered myocardial infarction before surgery (Figure 7).

### Calcification

We observed calcification in organized intracoronary thrombi as well as in coronary artery walls in patients without thrombosis. Single calcifications were detected using echocardiography two years after KD onset. Thereafter, calcification progressed to a severe stage in 5-7 years after KD onset in 2 patients with calcium score 1242, volume 952 mm^3^.

## Discussion

The patients with KD in the Moscow region had typical features of the disease described in the literature but the proportion of patients with coronary artery aneurysms was higher than reported from Japan and the United States^2^. We assume that this is due to delayed treatment, the use of low-dose intravenous immunoglobulin, and ascertainment bias with only the most severe patients being correctly diagnosed. It is likely that less severe cases of KD have gone undiagnosed. In some cases the diagnosis of KD was established only on the basis of coronary artery aneurysms detection.

Our observations confirm findings of other authors that patients with giant aneurysms have the highest risk of coronary artery obstruction^2,4,8–11^. Our data about the terms of formation, localization and outcomes of the intracoronary thrombi and obstruction of the coronary arteries correspond to the literature. Intracoronary thrombi in giant aneurysms were detected during the first two years after KS onset in all patients except one. More than a half of all these patients developed thrombi in the first 60 days.

According to our observations, even large intracoronary thrombi may regress when using conservative antithrombotic therapy. This avoids surgery in some patients with blood clots in coronary aneurysms without myocardial ischemia. On the other hand, timely surgery could have prevented myocardial infarction in patients with ineffective antithrombotic therapy. In our cohort 4 of the 5 patients with severe and critical stenosis presented no complaints and had no evidence of ischemia according to ECG and Holter monitoring.

There are also reports in the literature about the absence of signs of ischemia according to the exercise stress ECG in a patient with 99% stenosis of the LAD who suffered a fatal myocardial infarction soon after the examination^4^. Surgical treatment in 4 of our 5 patients prevented the myocardial infarction owing to the regular examinations, the use of different imaging techniques and cooperation of pediatricians and cardiac surgeons. We believe that such an approach can decrease the risk of irreversible changes in the myocardium.

Thus, over the last 13 years physicians’ awareness about KD increased in the Moscow region. As a result, there is a tendency to earlier diagnosis of KD, administration of an effective treatment corresponding to the international recommendations and reduction of the number of patients with coronary artery aneurysms. However, there are still many problems. There are still cases of late and wrong diagnosis that may lead to formation of coronary artery aneurysms including giant aneurysms. The internationally accepted dose of intravenous immunoglobulin is not always administered due to organizational and economic reasons. Not all patients with giant aneurysms receive antiplatelet drugs in combination with anticoagulants. The possibility of regular INR monitoring is not always present, increasing the risk of coronary thrombosis. The situation with timely diagnosis and treatment of KD in big cities is much better than in remote small settlements. So, we consider the educational work among doctors in such small settlements far away from large medical centers to be an important way to decrease the prevalence and severity of coronary artery lesions in patients with KD in our region.

Besides local complexities there are unsolved problems of diagnosis and treatment of KD in the world. Diagnosis of KD may be very difficult due to absence of pathognomonic signs and specific laboratory tests. Development of improved clinical and laboratory diagnostic algorithm could facilitate diagnosis by physicians who have no experience in establishing the diagnosis of KD^12^. All patients with a history of coronary artery aneurysms require long-term management to prevent coronary thrombosis, to detect severe stenosis of the coronary arteries, and prevent ischemia.

### Limitations

The retrospective method we used can cause some data distortion. One more limitation of the study, which has to be mentioned, is the small number of patients with giant aneurysms, coronary thrombosis, coronary stenosis. The duration of observation is short in this study and it is necessary to continue monitoring.
